# CannaCount: an improved metric for quantifying estimates of maximum possible cannabinoid exposure

**DOI:** 10.1038/s41598-023-32671-9

**Published:** 2023-04-11

**Authors:** Ashley M. Lambros, Kelly A. Sagar, M. Kathryn Dahlgren, Deniz Kosereisoglu, Celine El-Abboud, Rosemary T. Smith, Staci A. Gruber

**Affiliations:** 1 Cognitive and Clinical Neuroimaging Core (CCNC), McLean Hospital, 115 Mill St, Belmont, MA 02478 USA; 2 Marijuana Investigations for Neuroscientific Discovery (MIND) Program, McLean Hospital, 115 Mill St, Belmont, MA 02478 USA; 3 Department of Psychiatry, Harvard Medical School, 401 Park Drive, Boston, MA 02215 USA

**Keywords:** Psychology, Medical research, Toxicology

## Abstract

Increasing numbers of individuals have access to cannabinoid-based products containing various amounts of delta-9-tetrahydrocannabinol (THC), cannabidiol (CBD), and other cannabinoids. Exposure to specific cannabinoids likely influences outcomes; however, current methods for quantifying cannabis exposure do not account for the cannabinoid concentrations of the products used. We developed CannaCount, an examiner-driven metric that quantifies estimated maximum possible cannabinoid exposure by accounting for variables related to cannabinoid concentration, duration, frequency, and quantity of use. To demonstrate feasibility and applicability, CannaCount was used to quantify estimated maximum THC and CBD exposure in 60 medical cannabis patients enrolled in a two-year, longitudinal, observational study. Medical cannabis patients reported using a variety of product types and routes of administration. Calculating estimated exposure to THC and CBD was possible for the majority of study visits, and the ability to generate estimated cannabinoid exposure improved over time, likely a function of improved product labeling, laboratory testing, and more informed consumers. CannaCount is the first metric to provide estimated maximum possible exposure to individual cannabinoids based on actual cannabinoid concentrations. This metric will ultimately facilitate cross-study comparisons and can provide researchers and clinicians with detailed information regarding exposure to specific cannabinoids, which will likely have significant clinical impact.

## Introduction

Given the ongoing trend of legalization across the US, the majority of Americans have access to cannabis and products containing cannabinoids, including delta-9-tetrahydrocannabiol (THC), the primary intoxicating constituent of the plant, and cannabidiol (CBD), a primary non-intoxicating compound. Despite increased public accessibility, scientists often face obstacles when conducting cannabinoid-based research. In the US, despite expanded legalization efforts across states for medical and/or recreational use, multiple institutional approvals (i.e., Food and Drug Administration [FDA], Drug Enforcement Administration [DEA], and Institutional Review Boards [IRBs]) are needed to conduct clinical trials, and until recently the National Institution of Drug Abuse (NIDA) was the only approved source of cannabis for US-based clinical research studies. While researchers often avoid some regulatory obstacles by conducting observational studies of cannabis consumers, this approach has its own unique scientific challenges, including significant difficulty quantifying estimated cannabinoid exposure, which is in large part related to the heterogeneity of cannabis and cannabinoid-based products.

Traditionally, most research studies have examined individuals who use inhaled preparations of cannabis. In these studies, cannabis use is typically quantified by self-reported frequency of use (uses per day/week/month/year; lifetime uses) using traditional or modified timeline followback procedures^1,2^. However, focusing only on frequency fails to account for the quantity of cannabis used, an important predictor of the impact of cannabis use on various outcomes^3^. While some have used proxies for estimating quantity (e.g., number of puffs, joints/day), given the shortcomings of these methods, others have estimated quantity using self-reported amounts (e.g., grams, ounces)^4–14^ or by observing and measuring the amount of a substitute product (e.g., oregano) that cannabis consumers rolled into joints^15^, a method shown to be reliable, valid^16^, and a better predictor than simpler measures of estimated quantity^17^.

In recent years, methodologies that incorporate important details beyond frequency and amount of cannabis used have also been developed, including the Marijuana Smoking History Questionnaire (MSHQ)^18^ and the Daily Sessions, Frequency, Age of Onset, and Quantity of Cannabis Use Inventory (DFAQ-CU)^19^. However, as noted in a recent review^20^, current measures have significant limitations. For example, Wetherill and colleagues’^21^ proposed metric, which involves multiplying the number of grams consumed per day by years of cannabis use (“gram years”), only accounts for *inhaled* routes of administration (e.g., smoking or vaping) and does not include other modes of use. Others have recommended a “standard joint unit” (SJU) in which a joint is equated to a specific quantity (grams, puffs or hits)^3,16^ or amount of THC measured in mg or g (e.g., “SJU = 1 joint = 0.25 g of cannabis = 7 mg of THC”)^22^. However, none of these methods considers the variable amounts of THC or CBD across cultivars and products.

Overall, the increasing heterogeneity of cannabinoid-containing products makes it challenging for clinicians and researchers to accurately quantify exposure to specific cannabinoids. In an attempt to account for varying amounts of cannabinoids across cultivars, Hindocha et al.^23^ propose categorizing products based on THC and CBD ratios, suggesting that “1 standard cannabis unit equals 0.25 g of a variety with high tetrahydrocannabinol and low cannabidiol concentrations, 0.5 g of a variety with equal tetrahydrocannabinol and cannabidiol concentrations, and 0.75 g of a variety with low tetrahydrocannabinol and high cannabidiol concentrations.” However, utilizing fixed ratios results in a loss of precise data and increases inaccuracy, as ratios are not analogous to actual amounts of individual cannabinoids. For example, a product containing 50 mg CBD/25 mg THC and a product containing 4 mg CBD/2 mg THC both have a 2:1 ratio of CBD to THC but will likely confer very different effects. Further, extremely variable cannabinoid constituent profiles and concentrations exist across available products. Studies in several countries have shown that THC levels of cannabis flower have risen drastically in recent years^24–26^. In the US, analyses of government-seized cannabis revealed that average THC levels rose from 3.96% in 1995 to 17.10% in 2017, an increase of 332%^24,25^. In addition, the expanding legal cannabis market has brought about a proliferation of novel cannabis products, which have now become mainstream. Products come in many forms (e.g., flower, oils, concentrates, edibles, beverages, capsules/pills, topicals, suppositories), can be used in a variety of ways (e.g., inhalation, oral, oromucosal, cutaneous, transdermal, and transmucosal), and offer a wide range of cannabinoid constituents in varying concentrations^20^. Notably, concentrates (e.g., wax, shatter, budder, dabs) contain extremely high levels of THC. While a previous report noted that THC content can approach 80% in these products^27^, concentrates sold in dispensaries often list even higher THC content, with many exceeding 90%^28^. In contrast, hemp-derived products, created using low-THC varieties of cannabis (< 0.3% THC), have become extremely popular in recent years, particularly since 2018 when the Farm Bill was signed into law, effectively legalizing hemp in the US.

Importantly, additional challenges include lack of universal standards for cannabinoid product label information, discrepancies between product labels and laboratory-provided certificates of analyses (COAs) listing concentrations of individual cannabinoids^29–31^, variability of self-report data^32^, and changes in product use over time. Each of these factors impact the ability to quantify cannabis use, underscoring the need for comprehensive tools designed to generate more accurate quantifications of estimated exposure to cannabinoids in a systematic, standardized manner. Given the lack of consistency and shortcomings of current methodologies, we developed CannaCount, a metric that generates an estimated quantification of current or recent *maximum* possible cannabinoid exposure across a variety of product types and accounts for specific cannabinoid concentrations as well as frequency and quantity of each product used. Given that different routes of administration each impact bioavailability, CannaCount generates cannabinoid exposure for four separate routes of administration (inhaled, oral, mucosal, and skin-based applications). In addition, as a single value is often required when dealing with groups of heterogeneous cannabis consumers who use a wide range of products, an optional step is also included to allow for calculation of a single estimate of exposure across all routes of administration, generating an overall ‘maximum possible exposure’ for each individual cannabinoid. To demonstrate feasibility and applicability, we utilized CannaCount to generate estimated maximum THC and CBD exposure in a sample of medical cannabis patients enrolled in a longitudinal, observational study who reported using a variety of real-world cannabinoid-based products.

## Methods

### Metric variables

To accurately quantify maximum possible exposure to individual cannabinoids using CannaCount, several discrete pieces of information are required, including product type(s), route of administration, cannabinoid concentrations, and pattern of use for each product (duration, frequency, and quantity used over a specific interval of time). Additional information about each variable and general methods that can be used to acquire data are noted in Table 1. Further, Supplemental File 1 (“CannaCount Guide”) contains a guided interview comprised of specific questions that can be utilized to gather information about cannabis use (CannaCount Guide Step 1), as well as steps for completing calculations (CannaCount Guide Steps 2–8). Additional guidelines for collecting data on each of these variables and instructions for how to calculate estimates of maximum possible THC and CBD exposure (in mg) are provided below. An example of how to complete the interview and subsequent calculations is provided in Supplemental File 2.

**Table 1:** Information about key variables for cannabinoid calculations. CannaCount requires acquisition of data for several key variables; the specific information needed for each variable are provided along with suggestions for how to acquire these data.

Information needed about each product	Data needed	How to acquire data
Product type	Specify type as:	Timeline followback (TLFB)
	Flower	Drug diaries
	Concentrate	Other clinical interview (e.g., Supplemental File 1)
	Edible	
	Pill	
	Oil/tincture/solution	
	Lozenges/dissolvable products	
	Suppository	
	Oral spray	
	Topical	
	Transdermal Patch	
	Nasal spray	
Route of administration^a^	Specify route of administration as:	TLFB
	Inhalation (smoked, vaped, dabbed)	Drug diaries
	Oral (ingested)	Other clinical interview (e.g., Supplemental File 1)
	Mucosal (oromucosal, transmucosal)	
	Skin-based applications (cutaneous; transdermal)	
Cannabinoid constituent information	Determine mg of THC and CBD to appropriate unit	Laboratory cannabinoid quantification
	(see Supplemental files 1 & 2 for preferred units and for a complete guide to quantifying estimates of cannabinoid quantification and an example)	Certificates of analyses from manufacturers/dispensaries
		Product labels
Frequency	Determine frequency of use for each product (times per day, days per week, days per month, etc.)	TLFB
	Convert to uses per week (or other desired frequency)	Drug diaries
		Other clinical interview (e.g., Supplemental File 1)
Amount used	Determine amount per use of each product (g, ml, oz, # of servings, etc.)	TLFB
	OR	Drug diaries
	how long it takes individual to use a specified quantity (e.g., 10 ml in 7 days)	Other clinical interview (e.g., Supplemental File 1)
		May also utilize visual aids or ‘roll-a-joint’-like paradigms that are adapted to relevant mode of use
Duration used	Determine how long each product was used during the interval being assessed	TLFB
		Drug diaries
		Other clinical interview (e.g., Supplemental File 1)

^a^Note: although route of administration is data not used in cannabinoid exposure calculations, it can be useful for qualitative descriptions of cannabinoid product use across a sample.

#### Product type(s) & route(s) of administration

The total number of regularly used products during a specified interval of time should be ascertained, and product type and route of administration should be recorded for each product. Recommended classifications for product types and routes of administration are noted in Table 1. Cannabis consumers and patients typically try a variety of products, especially when they have access to recreational and/or medical dispensaries which offer wide selections of products that often change over time. Accordingly, it is not always feasible to calculate cannabinoid exposure for *all* products an individual has tried, and it is therefore recommended to account for any product(s) used “consistently” (i.e., products reported as being incorporated regularly into an individual’s regimen). Once regularly used products are identified, information regarding cannabinoid profile and pattern of use are necessary to calculate maximum possible cannabinoid exposure, as described below.

#### Cannabinoid concentrations

Cannabinoid concentrations can be acquired from several sources. The most accurate and unbiased quantification of cannabinoids comes from independent laboratory analyses which generate a constituent profile including an absolute quantification of individual cannabinoids present; this approach is also useful when constituent information is not readily available (e.g., unlabeled or homegrown products). While laboratory testing is currently the gold standard for ensuring accurate cannabinoid concentration information is acquired, utilizing COAs provided by manufacturers or vendors is a viable alternative when independent laboratory analyses are not possible, but COAs must match the batch of the product being assessed, as cannabinoid content varies across batches. Although not recommended, information from product labels could be used if product testing is not feasible and COAs are unavailable. Given reports of inaccurate product labeling, information from labels should be used sparingly and only as a proxy for more objective cannabinoid information when data from other sources cannot be obtained. It is always recommended to disclose when product labels are used as the source for cannabinoid concentration data and to note this as a limitation when publishing these data.

Often, additional details beyond what is reported in lab reports, COAs, and product labels, are also needed to generate actual mg of THC or CBD contained per specified unit, particularly when relying on product labels. Specifically, information including “serving size” or recommended dose, servings per container, or total volume of the container may also be required. When possible, cannabis consumers should share pictures of product labels and relevant packaging to assist with calculations.

It is also important to account for the conversion of cannabinoids from their acid forms to neutral or active forms (i.e., THC_A_ to THC, CBD_A_ to CBD) that occurs when products are decarboxylated. For heated products including flower, vape cartridges/pens, and concentrates, “Max THC” and “Max CBD” can be calculated using the equations provided in Step 4 of the CannaCount Guide, which are used by laboratories as part of their quantification process^33^.

#### Duration of use

For each product, the time period in which it was used regularly should be noted (e.g., start date and end date, number of weeks used) as well as the overall interval of time being assessed (e.g., examining cannabinoid use over the past 6 months).

#### Frequency of use

Determine the number of days per week, per month, or per any discrete time period that each product is used, as well as the number of times per day it is used.

#### Amount of product used

To estimate the actual amount of each product used*,* two methods can be utilized depending on how individuals best recall their cannabis use. The first option is to record the *average, discrete amount of product used each time* (e.g., grams/use, ml/use, number of servings/use). Alternatively, an individual may report *how long it takes to use the total quantity of a product* (e.g., 1 ml vape cartridge lasts 1 week, 2 g of flower lasts 4 days, a 30 ml bottle of tincture lasts 1 month). Importantly, as CannaCount has been developed to prioritize accuracy of recalling and reporting cannabis use, individuals may find it easiest to report their use of different products in different ways. Accordingly, individuals may use one or both methods to report amount of cannabis use; the way(s) an individual reports the amount of product(s) used will dictate the approach used for estimating exposure, as described below.

### Calculating THC and CBD exposure

After using the CannaCount guide (Steps 1–2) to gather information for each of the variables noted above (i.e., product type, cannabinoid concentrations, duration of time product is used, frequency, and amount of product used), THC and CBD exposure can be calculated using either the discrete use approach or the total use approach (see CannaCount guide Step 3).

#### Discrete use approach

The discrete use approach utilizes frequency, amount, and cannabinoid concentrations per product used, which is then multiplied by the total number of uses and averaged over the interval of use to estimate cannabinoid exposure. This approach is suitable for products with discrete content per use or serving (e.g., 1 g of flower contains *x mg* of THC and *x* mg of CBD; 1 capsule contains *x* mg of THC and *x* mg of CBD).

#### Total use approach

The total use approach utilizes the total cannabinoid content of each product used, which is then averaged over the time interval to estimate cannabinoid exposure. This method is less burdensome and preferred when patients are not using products with specific content noted per use/serving or cannot reliably estimate the amount of product they use each time. While it is strongly recommended to obtain an estimate of frequency of use, the total use approach does not require this information to calculate weekly (or monthly, yearly, etc.) cannabinoid exposure.

Once the approach (discrete vs. total use) for each product has been determined, calculations can be completed using Steps 4–7 of the CannaCount guide to estimate THC and CBD exposure separately for four routes of administration: inhaled, oral, mucosal (oromucosal and transmucosal), and skin-based applications (cutaneous and transdermal). When needed, an optional step 8 can also be implemented to generate a single metric of total maximum possible exposure for each cannabinoid. However, as route of administration significantly impacts the amount of bioactive drug “on board”, utilizing combined total estimates of cannabinoid exposures are likely less accurate than reporting by individual route of administration. Importantly, the inability to account for actual differences in bioavailability should be noted as a limitation whenever the combined, single metric of exposure is used and reported.

### Observational, longitudinal study data

#### Participants

To date, we have utilized CannaCount to calculate THC and CBD exposure in 60 medical cannabis patients who were recruited from the Greater Boston Metropolitan area and are currently enrolled in an ongoing observational, longitudinal study. This study was conducted in accordance with the Declaration of Helsinki and was approved by the Mass General Brigham IRB (2013P002611). Procedures, risks, benefits and the voluntary nature of the study were explained to patients, and written informed consent was obtained from all patients.

#### Data collection

As part of this two-year longitudinal study, data regarding use of cannabinoid-based products (product type, frequency, duration, quantity, cannabinoid concentrations) were collected by research staff during in-person study visits occurring every 3–6 months and during monthly phone check-ins occurring between study visits. Medical cannabis diaries were also used to corroborate data collected during study visits. THC and CBD exposure (per route of administration and overall) were then calculated for each subject for intervals between study visits using the described cannabinoid exposure metric. All calculations were reviewed and verified by a second staff member.

## Results

Medical cannabis patients reported using various product types and routes of administration, including inhalation (smoking or vaping flower, oils, concentrates; *n* = 37), oral (edibles, capsules/tablets; *n* = 29), mucosal (sublingual solutions, lozenges; *n* = 42), and skin-based applications (“topical” lotions, balms, etc.; *n* = 8). Using frequency of use, amount used, duration of use and cannabinoid concentrations for each of these products, overall THC and CBD exposure were independently calculated for 150 of the 190 visits conducted (79.0%). Missing data were almost exclusively limited to the early stages of the study prior to development of CannaCount, which in fact helped shape the current methods described; in the most recent years (2018–2021), quantification was possible for 93.9% of visits. Cannabinoid use data for the current sample is provided in Table 2**,** which includes maximum possible exposure for each route of administration as well as an estimate of total maximum possible exposure for all products.

**Table 2:** Cannabinoid use characteristics. To demonstrate feasibility and applicability of CannaCount, averages and standard deviations are provided for frequency of use and cannabinoid exposure using data from 150 total study visits (*n* = 60 medical cannabis patients enrolled in an ongoing, longitudinal study).

Cannabinoid use variable	Mean (SD)
Frequency of use	
Days per week	5.95 (1.67)
Times per day	1.62 (0.89)
Total uses per week	9.99 (6.73)
Cannabinoid exposure	
Average THC exposure (mg/week)	
THC—inhaled^1^	68.24 (111.97)
THC—oral^2^	42.55 (147.45)
THC—mucosal^3^	10.33 (15.25)
THC—skin-based applications^4^	5.11 (9.99)
Overall THC exposure^5^	43.29 (110.37)
Average CBD exposure (mg/week)	
CBD—inhaled^6^	48.39 (111.19)
CBD—oral^7^	37.33 (54.29)
CBD—mucosal^8^	214.90 (297.63)
CBD—skin-based applications^4^	2.22 (1.46)
Overall CBD exposure^5^	166.69 (258.41)

^1^*n* = 52.

^2^*n* = 46.

^3^*n* = 92.

^4^*n* = 5.

^5^*n* = 150.

^6^*n* = 51.

^7^*n* = 45.

^8^*n* = 97.

## Discussion

CannaCount was developed to provide a standardized tool for calculating and quantifying estimated maximum possible exposure to individual cannabinoids, which helps address challenges regarding accurate estimation of cannabinoid exposure. Unlike previous methods, CannaCount utilizes information about specific cannabinoid concentrations, which is then combined with information about product type, frequency and amount of use. CannaCount accounts for changes in cannabinoid use over time, ultimately generating an estimate of the actual mgs of THC and CBD that an individual is exposed to over a given period. This comprehensive approach represents a considerable improvement over previous methods, which are based solely on estimated frequency and/or amount of cannabis used, and even those that attempt to consider average ratios of THC and CBD in cannabis^23,34^. Calculating accurate estimates of exposure will help clarify the impact of cannabinoids on treatment outcomes, generate more accurate risk/benefit ratios, and aid in assessing potential for drug-drug interactions between cannabinoid-based products and other medications or substances.

While CannaCount requires collecting data from multiple sources and may be more time consuming than other methods, we have successfully implemented this approach in several clinical research studies. To demonstrate feasibility, we estimated THC and CBD exposure using this metric in 60 medical cannabis patients enrolled in an ongoing, longitudinal study who used a wide variety of products. As noted, information required for calculating THC and CBD exposure was available for 150 of the 190 (78.95%) patient visits completed at the time of analyses. Notably, this study began soon after medical cannabis was legalized in Massachusetts when few labs were able to complete full cannabinoid constituent analyses and product labels were often incomplete, resulting in missing data. Over time, our ability to quantify cannabinoid exposure increased dramatically as CannaCount was developed and implemented. Specifically, over the first four years of the study (2014–2017), cannabinoid quantification was only achieved for 44.83% of visits, while in the most recent years (2018–2021), quantification has been possible for 93.94% of visits. This improvement, which highlights the feasibility of CannaCount, is likely related to improved cannabinoid product labeling, laboratory testing of patients’ products, and more informed consumers.

As THC and CBD are the two most abundant cannabinoids and most commonly reported on product labels, we focused exclusively on THC and CBD. Importantly, however, CannaCount can be used to calculate exposure to *any* cannabinoid—but only if the actual amount of each minor cannabinoid within a given product is known and verified via lab analyses. The potential ability to calculate exposure to minor cannabinoids is an important advantage of this tool given the recent proliferation of products featuring minor cannabinoids, and is particularly important as cannabis is often treated as “one thing,” even though individual cannabinoids exert unique effects.

Despite addressing several challenges that currently prevent researchers and clinicians from accurately estimating cannabinoid exposure, some limitations remain. Estimated exposure was not possible for approximately 21% of patient visits over the eight years of the study, primarily due to limited product label information early on in the study. Notably, however, for all patients whose cannabinoid exposure could not be quantified, overall frequency of use was calculated. This information can be used as an ancillary variable for analyses, particularly when estimates of THC and CBD exposure cannot be determined for the entire sample, and could also be helpful information for clinical decision-making.

While information about actual cannabinoid concentrations is the most important for accurately estimating cannabinoid exposure, it is also the most challenging data to collect. Laboratory analyses of cannabinoid products should be prioritized given their specificity and accuracy. It is also important, however, to acknowledge that these analyses are often cost prohibitive and require patients or research participants to submit a sample of their product(s) which is not always feasible; individuals may not have product available to submit or may be unwilling to submit especially if they are not compensated. As a result, availability of individual products for laboratory testing may differ across study populations and impact feasibility of CannaCount. Notably, while some companies provide COAs for some of their products, this practice is not universal, and it is important to ensure that COAs “match the batch” for the actual product used by the patient. Further, although product labels are the most convenient source of information, several studies demonstrate that labels are often inaccurate, and many do not consistently list the presence of all cannabinoids (e.g., hemp-derived products may not report THC content, even when present)^29–31,35,36^. Moreover, clearly defined serving sizes for products containing multiple servings are not always on the label^37^. Accordingly, use of product label information is not recommended and should only be used if constituent information from more objective sources is not available. While CannaCount may require additional time relative to standard questionnaires, data generated is likely more accurate and comprehensive than existing methods, and both feasibility and accuracy of this metric will likely improve over time if regulations to standardize product labels are enacted. Ideally, with growing recognition of the issues related to inaccurate labeling of cannabinoid products, future legislation will address requirements and guidelines for product labeling, promoting better label accuracy in the coming years and ultimately providing researchers with a more cost-effective method for generating accurate cannabinoid exposure calculations.

In addition, there are no universal standards for reporting cannabinoid concentrations in lab reports (including COAs) or on product labels. Some product labels include information about the amount of cannabinoids contained per serving, while others may list total cannabinoids in the package. Different units of measure are often reported (g, mg, ml, oz, etc.). As a result, those querying cannabinoid use and quantifying exposure must ensure they collect all necessary details, including cannabinoid content, serving size, number of servings per packages, and total weight or volume of the product. It is recommended that photographs of product packaging and labels are taken to aid in calculations. In the future, these issues may be remediated with laws that require standardized packaging and labeling of cannabinoid-based products^35^, such as requiring that quantities of THC, CBD, and a range of other common cannabinoids are included per serving, and that serving size and number of servings per package are noted. Requiring manufacturers to a) routinely test products using independent laboratories and b) make COAs accessible would simultaneously educate patients and consumers about their products and facilitate accurate cannabinoid quantification.

As with other research studies which rely on patient or participant-generated information, the accuracy of self-report data remains a limiting factor. A previous study found that single cannabis use estimates tend to be overestimated, but more macro-level estimates (i.e., 5 g used in one week) are likely to be more accurate ^38^. For the current metric, an examiner-driven approach to data collection is highly recommended. Although this approach still relies on self-report data, the examiner can corroborate and verify estimates by asking about use in various ways (i.e., “how much do you use each time?”, “how much did you buy?”, and “how long did it take you to use it?”). It is also recommended to incorporate methods designed to facilitate accurate self-reporting, such as visual aids to assist in estimation of quantity consumed per use and have individuals utilize daily or weekly cannabis use diaries. It is also of note that self-reports of cannabis use may be more accurate in certain populations; for example, medical cannabis patients who have a set treatment regimen or research participants who utilize drug diaries may be more likely to provide more accurate self-reports than adult/recreational users who use socially or sporadically. In addition, self-reported cannabis use may be affected by reticence to accurately report use given the legal status of cannabis and associated stigma. As liberal cannabis policies are associated with de-stigmatization^39^, widespread legalization or decriminalization will likely facilitate more honest and accurate self-reports over time.

Despite many advantages, including the ability to employ this metric in a population using a variety of product types and routes of administration, it is of note that the CannaCount approach reflects an estimate of *maximum* possible cannabinoid exposure and does not account for differences in bioavailability across different routes of administration^40,41^. Accordingly, estimated cannabinoid exposure is not equivalent to the exact amount of cannabinoids “on board.” As a current paucity of pharmacokinetic data limit our ability to accurately account for bioavailability in the formulae, CannaCount recommends calculating the maximum possible exposure for four discrete routes of administration; any use of the optional ‘single metric’ of total cannabinoid exposure in research studies should be disclosed as a limitation. Additional research is needed to determine average absorption across different product types, which is particularly important in light of increasing interest in using biospecimens to quantify cannabis use.

## Conclusions

An accurate, reliable method for quantifying estimated cannabinoid exposure is critical for clarifying the relationship between cannabinoid use and various outcomes measures. Establishing robust and standardized approaches for quantification will facilitate cross-study comparisons and ultimately help researchers and clinicians disentangle the unique effects associated with use of specific cannabinoids or cannabinoid-based products. CannaCount is the first metric to provide an estimate of maximum possible exposure to individual cannabinoids based on comprehensive product use data and represents a considerable improvement over previous approaches that do not account for variability in exposure to individual cannabinoids.

## Supplementary Information

Supplementary Information.

## Data Availability

The datasets used in the current study are not publicly available as they were used to demonstrate feasibility of the metric and will be utilized in future, planned analyses; however, data are available from the corresponding author on reasonable request.
