# Supplementary material for: CannaCount: an improved metric for quantifying estimates of maximum possible cannabinoid exposure
Source: Sci Rep. 2023 Apr 11;13:5869. doi: 10.1038/s41598-023-32671-9 (PMC10090150; doi:10.1038/s41598-023-32671-9)
Supplement: Supplementary file 1 — Supplementary Information. [file 41598_2023_32671_MOESM1_ESM.pdf]

SUPPLEMENTAL FILES

CannaCount: an improved metric for quantifying estimates of maximum possible cannabinoid exposure

Ashley M. Lambros, Kelly A. Sagar, M. Kathryn Dahlgren, Deniz Kosereisoglu, Celine El-Abboud, Rosemary T. Smith, & Staci A. Gruber

A Guide to Quantifying Estimates of Cannabinoid Exposure Using CannaCount

Step 1: Query Cannabinoid Use for Quantification

For this guide, instructions for the interviewer are noted in plain font. Questions to be asked aloud are noted in ***bold and italicized*** font.

For these questions, the term cannabis refers to any kind of marijuana, hemp, cannabidiol (CBD), or other cannabinoid-based products. Do you have any questions?

A. PAST USE (OPTIONAL)
Note: These questions are designed to establish rapport and to collect history of previous cannabis use, if needed.

1. ***Have you ever tried cannabis?***

   ☐ No *(discontinue interview)*

   ☐ Yes *(continue)*

2. ***How old were you the first time you ever tried cannabis?***

3. ***After the first time you tried cannabis, was there ever a period of time when you used it regularly, as in a consistent pattern of use such as every week or every month?***

   ☐ No

   a. ***How many times have you tried cannabis?***

   ☐ Yes

   a. ***How old were you when you began using regularly?***

   b. ***At that time, how often were you using?***

   c. ***At that time, how much were you using?***

   d. ***At that time, were you using cannabis recreationally (to feel altered or high), for medical purposes (to address symptoms), or both?***

   e. **Optional Question:** ***What products were you using at that time?***

B. CURRENT USE

Record the interval of time being assessed for estimated cannabinoid exposure:

Start date: ___________ End date: ___________ = _________________________________
[total duration of time, including end date]

General information

This section does not provide information for calculating estimates of cannabinoid exposure.

1. ***When was the last time you used cannabis?***

2. ***Do you currently use cannabis recreationally (to feel altered or high), for medical purposes (to address symptoms), or both?***

3. ***On average, how often do you use any cannabis products? (i.e., per day, week, month, etc.)***

Cannabinoid Product information

Ask the following questions to collect information about an individual's current product regimen. Data will be organized and used for calculating estimated cannabinoid exposure.

4. Product Name/Type and Route of Administration:
   Note: See Supplemental Table 1 classification suggestions for cannabinoid products and routes of administration, as well as information regarding units of measure for cannabinoid constituents.

   a. ***How many cannabis products have you consistently used between [insert start date] and [insert end date]?***

      Note: Consistent use can be interpreted as any established pattern of use (e.g., 1 use per *x* period of time); for research purposes, a set definition of "consistent" should be established

   b. ***Can you tell me the name and type of each cannabis product?***
      Record name and type of consistently used products.

   c. For products with multiple options for route of administration, ask:
      How do you use [product name]?

      Note: If necessary, provide examples. (e.g., individual reports using a tincture. "How do you use [product name]? Is it added to food? Swallowed? Held under tongue for *x* period of time and then swallowed?")

ASK QUESTIONS 5-7 FOR EACH CURRENTLY USED CANNABINOID PRODUCT

Between [start date] and [end date]…

5. Duration, Frequency, and Amount:
   a. ***…when did you start using [product name]? Are you still currently using [product name]?*** If no, ***when did you stop using [product name]? Were there any periods of time when you did not use [product name]?***
   Record start date, end date, or date of interview if still currently using – estimates are acceptable.

   b. ***…on average, how many days per week or month do you use [product name]?***
   Record days per week or days per month

   c. ***…on days you use [product name], how many times per day do you use it?***
   Record times per day

   d. ***…how much of [product name] do you use each time you use it?***
   Record amount of product per use as well as cannabinoid constituent information per use if available

   If a quantifiable amount is not provided, reframe question:

   If finished the product: ***How much did you buy of [product name] and how long did it take for you to finish it?***

   If currently using but hasn't finished product: ***When did you buy [product name] and how much do you have left?***

6. Source of cannabinoid information:
   a. ***For [product name], can you provide the following?***
      i. ***Picture of the product label and packaging***
      ii. ***Link to manufacturer website***
      iii. ***Copy of the Certificate of Analysis (COA)***
   Note: Before completing the interview, ensure you have recorded the cannabinoid constituent information per use/amount/dose AND/OR for entire product

7. Independent lab analyses (optional):
   a. ***Would you be willing to submit a sample of [product name] to a lab for analysis of the cannabinoid content of your product?***
   Note: Ask regardless of information provided in 6a. Testing labs vary with regard to how much of each product type is required for cannabinoid constituent analyses.

Supplemental Table 1. Common Product Types, Routes of Administration, and Units for Reporting Cannabinoid Content

Product Type	Route of Administration	Common Units for Reporting Cannabinoid Content
Flower (Smoked, Vaped)	Inhalation	**mg/g**, %
Concentrates and Oils (Smoked, Vaped, Dabbed)	Inhalation	**mg/g** (solid forms), **mg/ml** (liquid forms), %
Edibles (Food or Beverages)	Oral	**mg/serving, mg/package**, **mg/container**
Pill (Capsule, Tablet, Softgel)	Oral	**mg/pill**, mg/serving, **mg/container**
Oils, Tinctures, Solutions	Muscosal (Oromucosal)	**mg/ml**, **mg/container**, mg/serving, %
Dissolvable product (Lozenge, Tablet, Strip)	Muscosal (Oromucosal)	Mg/package, **mg/serving**
Oral Spray	Muscosal (Oromucosal)	**mg/serving**, **mg/package**
Suppository	Muscosal (Transmucosal)	**mg/suppository**
Nasal Spray	Muscosal (Transmucosal)	**mg/serving**, **mg/package**
Topical (Lotion, Cream, Balm, Salve)	Skin-based applications (Cutaneous)	**mg/ml**, mg/fl oz, **mg/container**
Transdermal Patch	Skin-based applications (Transdermal)	**mg/patch**

Note: **Bolded** font indicates preferred unit for calculations
Preferred units for discrete use approach: mg/ml, mg/serving, or mg/pill
Preferred units for total use approach: mg/package or mg/container

Step 2: Organize Product Regimen Data Collected During Interview

Specifically, fill out the table below using responses from questions 4-6

Route of Administration*	Product Type	Frequency	Duration	Amount	Constituent Information (Include cannabinoid content with unit of measure & source of information)
☐ Inhalation	☐ Smoked flower<br>☐ Vaped flower<br>☐ Smoked concentrate<br>☐ Vaped oil/pen<br>☐ Vaped/dabbed concentrate				
☐ Oral	☐ Edible (food, beverage)<br>☐ Pill (capsule, tablet, soft gel)				
Mucosal:<br>☐ Oromucosal<br><br><br><br>☐ Transmucosal	☐ Oil, tincture, solution<br>☐ Dissolvable product (lozenge, tablet, strip)<br>☐ Oral spray<br>☐ Vaginal suppository<br>☐ Rectal suppository<br>☐ Nasal spray				
Skin-based applications:<br>☐ Cutaneous<br><br>☐ Transdermal	☐ Topical (lotion, cream, balm, salve)<br><br>☐ Transdermal patch				

**Note: Although route of administration data not used in cannabinoid exposure calculations, it can be useful for qualitative descriptions of cannabinoid product use across a sample*

Step 3: Review Frequency and Amount of Use Data & Determine Calculation Approach

Using information provided about use, determine which approach to use for each product:

- Discrete Use Approach:
    - Use when an individual reports how frequently they use a product, and the discrete amount of product used each time
    - **Examples of frequency:**
        - *x* uses per day, week, or month
        - *x* uses per day, and used *x* days per week or month
    - **Examples of amount per use:**
        - *x* grams of flower, wax, etc.
        - *x* ml of oil
        - *x* number of pills, gummies, etc.
        - *x* number of servings, doses

- Total Use Approach:
    - Use when an individual reports the total amount of product they use in a given time period
    - **Examples of total amount:**
        - *x* g or oz of flower
        - *x* ml or g of vape cartridge
        - *x* mL bottles of solution, x bottles in total
        - *x* fl mL or oz bottle of lotion
    - **Examples of time periods:**
        - x number of days, weeks, OR months

Step 4: Convert Cannabinoid Information to Preferred Units (if necessary)

- <u>Flower, vape cartridge OR other inhaled products:</u>
  - If necessary, convert mL vape cartridge to g (e.g., 1000mL = 1g or 500mL = 0.5g)
  - If necessary, convert % THC and %CBD to mg/g
    - Multiple percentage by 10 (e.g., 1% = 10mg/g)
  - Convert THC & THC_A to Max THC
    - *Max THC (mg) = (0.877 x THC_A mg/g) + THC mg/g*
  - Convert CBD & CBD_A to Max CBD
    - *Max CBD (mg) = (0.877 x CBD_A mg/g) + CBD mg/g*

- <u>Oil, tincture, solutions OR topicals:</u>
  - If necessary, convert mg/fl oz to mg/ml (*1 fl oz = 29.57ml)*

**Organize Final THC and CBD content:**

Step 5: DISCRETE USE Approach - Calculate THC and CBD Exposure

<u>Concept:</u> Average THC or CBD Exposure = (cannabinoid content x amount used x frequency of use x duration of use)/time frame

<u>Note:</u> Choose a formula to calculate THC and CBD exposure for each product, or adjust to desired time frame

a. Calculate THC exposure for each product:

<u>Per day formula:</u> Average THC exposure (mg/day) = $\frac{\text{mg of THC}}{\text{unit (g,mg,ml,etc.)}} \times \frac{\text{amount (g,mg,ml,etc.)}}{\text{use}} \times \frac{\text{\# of uses}}{\text{day}} \times \frac{\text{\# of days used}}{\text{total number of days in interval}}$

<u>Per week formula:</u> Average THC exposure (mg/week) = $\frac{\text{mg of THC}}{\text{unit (g,mg,ml,etc.)}} \times \frac{\text{amount (g,mg,ml,etc.)}}{\text{use}} \times \frac{\text{\# of uses}}{\text{day}} \times \frac{\text{\# of days used}}{\text{week}} \times \frac{\text{\# of weeks product used}}{\text{total \# of weeks in interval}}$

<u>Per month formula:</u> Average THC exposure (mg/month) = $\frac{\text{mg of THC}}{\text{unit (g,mg,ml,etc.)}} \times \frac{\text{amount (g,mg,ml,etc.)}}{\text{use}} \times \frac{\text{\# of uses}}{\text{day}} \times \frac{\text{\# of days used}}{\text{month}} \times \frac{\text{\# of months product used}}{\text{total \# of months in interval}}$

b. Calculate CBD exposure for each product:

<u>Per day formula:</u> Average CBD exposure (mg/day) = $\frac{\text{mg of CBD}}{\text{unit (g,mg,ml,etc.)}} \times \frac{\text{amount (g,mg,ml,etc.)}}{\text{use}} \times \frac{\text{\# of uses}}{\text{day}} \times \frac{\text{\# of days used}}{\text{total number of days in interval}}$

<u>Per week formula:</u> Average CBD exposure (mg/week) = $\frac{\text{mg of CBD}}{\text{unit (g,mg,ml,etc.)}} \times \frac{\text{amount (g,mg,ml,etc.)}}{\text{use}} \times \frac{\text{\# of uses}}{\text{day}} \times \frac{\text{\# of days used}}{\text{wk}} \times \frac{\text{\# of weeks product used}}{\text{total \# of weeks in interval}}$

<u>Per month formula:</u> Average CBD exposure (mg/month) = $\frac{\text{mg of CBD}}{\text{unit (g,mg,ml,etc.)}} \times \frac{\text{amount (g,mg,ml,etc.)}}{\text{use}} \times \frac{\text{\# of uses}}{\text{day}} \times \frac{\text{\# of days used}}{\text{month}} \times \frac{\text{\# of months product used}}{\text{total \# of months in interval}}$

Step 6: TOTAL USE Approach - Calculate THC and CBD Exposure

Concept: *Average THC or CBD Exposure = cannabinoid content x (total amount used/time frame)*

Note: Choose *one* formula to calculate THC and CBD exposure for each product, or adjust to desired time frame.

a. Calculate THC exposure for each product:

Per day formula: **Average THC exposure (mg/day) =** $\frac{\text{mg of THC}}{\text{unit (g,mg,ml,etc)}} \times \frac{\text{total amount used during interval (g,mg,ml,etc.)}}{\text{total \# of days in interval}}$

Per week formula: **Average THC exposure (mg/week) =** $\frac{\text{mg of THC}}{\text{unit (g,mg,ml,etc)}} \times \frac{\text{total amount used during interval (g,mg,ml,etc.)}}{\text{total \# of weeks in interval}}$

Per month formula: **Average THC exposure (mg/month) =** $\frac{\text{mg of THC}}{\text{unit (g,mg,ml,etc)}} \times \frac{\text{total amount used during interval (g,mg,ml,etc.)}}{\text{total \# of months in interval}}$

b. Calculate CBD exposure for each product:

Per day formula: **Average CBD exposure (mg/day) =** $\frac{\text{mg of CBD}}{\text{unit (g,mg,ml,etc)}} \times \frac{\text{total amount used during interval (g,mg,ml,etc.)}}{\text{total \# of days in interval}}$

Per week formula: **Average CBD exposure (mg/week) =** $\frac{\text{mg of CBD}}{\text{unit (g,mg,ml,etc)}} \times \frac{\text{total amount used during interval (g,mg,ml,etc.)}}{\text{\# of weeks in interval}}$

Per month formula: **Average CBD exposure (mg/month) =** $\frac{\text{mg of CBD}}{\text{unit (g,mg,ml,etc)}} \times \frac{\text{total amount used during interval (g,mg,ml,etc.)}}{\text{total \# of months in interval}}$

Step 7: Sum Exposure for Products within Each Route of Administration

<u>Concept</u>: Average THC or CBD Exposure= sum of the average cannabinoid exposure for each route of administration

a. Calculate THC exposure for each route of administration (inhaled, oral, mucosal, and skin-based applications)

<u>Formulae:</u>

THC Exposure for **Inhaled** Products: $THC_{Inhaled\ 1} + THC_{Inhaled\ 2} + THC_{Inhaled\ 3} ... + THC_{Inhaled\ n}$

THC Exposure for **Oral** Products: $THC_{Oral\ 1} + THC_{Oral\ 2} + THC_{Oral\ 3} ... + THC_{Oral\ n}$

THC Exposure for **Mucosal** Products: $THC_{Mucosal\ 1} + THC_{Mucosal\ 2} + THC_{Mucosal\ 3} ... + THC_{Mucosal\ n}$

THC Exposure for **Skin-based** Applications: $THC_{Skin\ 1} + THC_{Skin\ 2} + THC_{Skin\ 3} ... + THC_{Skin\ n}$

b. Calculate CBD exposure for each route of administration (inhaled, oral, mucosal, and skin-based applications)

<u>Formulae:</u>

CBD Exposure for **Inhaled** Products: $CBD_{Inhaled\ 1} + CBD_{Inhaled\ 2} + CBD_{Inhaled\ 3} ... + CBD_{Inhaled\ n}$

CBD Exposure for **Oral** Products: $CBD_{Oral\ 1} + CBD_{Oral\ 2} + CBD_{Oral\ 3} ... + CBD_{Oral\ n}$

CBD Exposure for **Mucosal** Products: $CBD_{Mucosal\ 1} + CBD_{Mucosal\ 2} + CBD_{Mucosal\ 3} ... + CBD_{Mucosal\ n}$

CBD Exposure for **Skin-based** Applications: $CBD_{Skin\ 1} + CBD_{Skin\ 2} + CBD_{Skin\ 3} ... + CBD_{Skin\ n}$

OPTIONAL Step 8: Sum Exposure Across All Products

<u>Concept</u>: Total Average THC or CBD Exposure= sum of the average cannabinoid exposure for all products used

a. Calculate THC exposure for all products

<u>Formula:</u> Total THC Exposure = $THC_{Inhaled} + THC_{Oral} + THC_{Mucosal} + THC_{Skin}$

b. Calculate CBD exposure for all products

<u>Formula:</u> Total CBD Exposure = $CBD_{Inhaled} + CBD_{Oral} + CBD_{Mucosal} + CBD_{Skin}$

Disclaimer*: Step 8 is included for instances where a single metric of cannabinoid exposure is needed. It is important to disclose when using this overall estimate of total exposure, particularly if multiple routes of administration are used, as differences in bioavailability are not accounted for, likely rendering this calculation less accurate than individual estimates of exposure for each route of administration (which are calculated in Step 7).*

Note*: To calculate estimates of total cannabinoid exposure across a group of patients or participants, use Step 8 to calculate total exposure for each individual first; then calculate the average cannabinoid exposure across the group. Do <u>NOT</u> calculate group averages in step 7 and sum exposure for each route of administration to generate total average cannabinoid exposure.*

A Guide to Quantifying Estimates of Cannabinoid Exposure Using CannaCount – Example

Step 1: Query Cannabinoid Use for Quantification

For this guide, instructions for the interviewer are noted in plain font. Questions to be asked aloud are noted in ***bold and italicized*** font. Example is written in blue ink.

For these questions, the term cannabis refers to any kind of marijuana, hemp, cannabidiol (CBD), or other cannabinoid-based products. Do you have any questions?

PAST USE (OPTIONAL)
Note: These questions are designed to establish rapport and to collect history of previous cannabis use, if needed.

1. ***Have you ever tried cannabis?***

   ☐ No *(discontinue interview)*

   ☑ Yes *(continue)*

2. ***How old were you the first time you ever tried cannabis?*** 16

3. ***After the first time you tried cannabis, was there ever a period of time when you used it regularly, as in a consistent pattern of use such as every week or every month?***

   ☐ No

   a. ***How many times have you tried cannabis?***

      NA

   ☑ Yes

   a. ***How old were you when you began using regularly?***

      18

   b. ***At that time, how often were you using?***

      2x/week

   c. ***At that time, how much were you using?***

      1g/week of flower

   d. ***At that time, were you using cannabis recreationally (to feel altered or high), for medical purposes (to address symptoms), or both?***
      Recreationally

   e. **Optional Question: What products were you using at that time?**
      Flower - cultivar unknown

B. CURRENT USE

Record the time period being assessed for calculating estimates of cannabinoid exposure:

Start date: _12/1/2021 End date: 2/22/2022 = ________12.00 weeks________________
[total duration of time, including end date]

General information

This section does not provide information for calculating estimates of cannabinoid exposure.

1. ***When was the last time you used cannabis?*** Yesterday evening at 10pm

2. ***Do you currently use cannabis recreationally (to feel altered or high), for medical purposes (to address symptoms), or both?*** Medical purposes

3. ***On average, how often do you use any cannabis products? (i.e., per day, week, month, etc.)*** Daily – sometimes uses multiple products per day

Cannabinoid Product information

Ask the following questions to collect information about an individual's current product regimen. Data will be organized and used for calculating estimated cannabinoid exposure.

4. Product Name/Type and Route of Administration:
   Note: See Supplemental Table 1 classification suggestions for cannabinoid products and routes of administration, as well as information regarding units of measure for cannabinoid constituents.

   a. ***How many cannabis products have you consistently used between [insert start date] and [insert end date]?***
   5 products

   Note: Consistent use can be interpreted as any established pattern of use (e.g., 1 use per *x* period of time); for research purposes, a set definition of "consistent" should be established

   b. ***Can you tell me the name and type of each cannabis product?***
   Flower 1, Flower 2 –smokes or vapes
   Oil 1, Oil 2 –takes by mouth
   Capsules – swallowed

   c. For products with multiple options for route of administration, ask:
   How do you use [product name]?
   Oil 1 and 2 – both are held under tongue for ~30 seconds before being swallowed (oromucosal)

   Note: If necessary, provide examples. (e.g., individual reports using a tincture. "How do you use [product name]? Is it added to food? Swallowed? Held under tongue for *x* period of time and then swallowed?")

ASK QUESTIONS 5-7 FOR EACH CURRENTLY USED CANNABINOID PRODUCT

Between [start date] and [end date]…

5. Duration, Frequency, and Amount:
   a. ***…when did you start using [product name]? Are you still currently using [product name]?*** If no, ***when did you stop using [product name]? Were there any periods of time when you did not use [product name]?***
   Flower 1 – using at start of interval (12/1), stopped 1/7
   Flower 2 – using at start of interval (12/1), stopped 1/7
   Oil 1 – started (1/14) and still using at end of interval
   Oil 2 – used throughout entire 12-week time period
   Capsules – 30 days

   b. ***…on average, how many days per week or month do you use [product name]?***
   Flower 1 or Flower 2 – 1 day/week, participant reported using both products interchangeably: Flower 1 75% of the time and Flower 2 25% of the time
   Oil 1 – 7 days/week
   Oil 2 – participant reported use varied, hard to estimate
   Capsules – 7 days/week

   c. ***…on days you use [product name], how many times per day do you use it?***
   Flower 1 – 1x
   Flower 2 – 1x
   Oil 1 – 2x
   Oil 2 – participant reported use varied, hard to estimate
   Capsules – 1x

   d. ***…how much of [product name] do you use each time you use it?***
   Flower 1 and Flower 2 – 0.25g per use
   Oil 1 – 0.5mL per use
   Oil 2 – not sure
   Capsules – 1 bottle (30 count)

   If a quantifiable amount is not provided, reframe question:

      If finished the product: ***How much did you buy of [product name] and how long did it take for you to finish it?***
      Oil 2 – used 2 100 mL bottles and currently halfway through bottle 3 (total = about 250 mL) during 12-week time period

      If currently using but hasn't finished product: ***When did you buy [product name] and how much do you have left?*** NA

6. <u>Source of cannabinoid information:</u>
    a. ***For [product name], can you provide the following?***
        i. ***Picture of the product label and packaging***
        Flower 2 and Oil 2

        ii. ***Link to manufacturer website***
        Oil 1 – COA provided by manufacturer

        iii. ***Copy of the Certificate of Analysis (COA)***
        Flower 1 and Capsule (see below)

    Note: Before completing the interview, ensure you have recorded the cannabinoid constituent information per use/amount/dose AND/OR for entire product

7. <u>Independent lab analysis (optional):</u>
    a. ***Would you be willing to submit a sample of [product name] to a lab that will analyze the cannabinoid content of your product?***
    Note: Ask regardless of information provided in 6a. Testing labs vary with regard to how much of each product type is required for cannabinoid constituent analyses.

    Yes – samples of Flower 1 and Capsule will be submitted for analyses

Step 2: Organize Product Regimen Data Collected During Interview

Specifically, fill out the table below using responses from questions 4-6

Route of Administration*	Product Type	Frequency	Duration	Amount	Constituent Information (Include unit & source)
☑ Inhalation	☑ Smoked flower<br>☑ Vaped flower<br>☐ Smoked concentrate<br>☐ Vaped oil/pen<br>☐ Vaped/dabbed concentrate	1x/week total:<br><br>Flower 1: 75% of the time<br><br>Flower 2: 25% of the time	Dec 1 – Jan 7 = 5.42 weeks	0.25 g per use	Flower 1 (source: lab report)<br>THC: 0mg/g THC, 209mg/g THC_A<br>CBD: 10mg/g CBD, 10mg/g CBD_A<br><br>Flower 2 (source: product label)<br>THC: 0.4% THC, 21.3% THC_A<br>CBD: 0.1% CBD, 0% CBD_A
☑ Oral	☐ Edible (food, beverage)<br>☑ Pill (capsule, tablet, soft gel)	1x/day	30 days = 4.28 weeks	1 30-count bottle (1 pill/day)	Capsule (source: lab report)<br>THC: 0.3 mg/capsule<br>CBD: 8.1 mg/capsule
Mucosal:<br>☑ Oromucosal<br><br>☐ Transmucosal	☑ Oil, tincture, solution<br>☐ Dissolvable product (lozenge, tablet, strip)<br>☐ Oral spray<br>☐ Vaginal suppository<br>☐ Rectal suppository<br>☐ Nasal spray	Oil 1: 2x/day<br><br>Oil 2: NA	Oil 1:<br>Jan 14 – Feb 22 = 5.71 weeks<br><br>Oil 2:<br>Dec 1 – Feb 22 = 12 weeks	Oil 1:<br>0.5 mL per use<br><br>Oil 2:<br>2.5 bottles (100 mL each)	Oil 1 (source: manufacturer COA)<br>THC: 1.63 mg/ml<br>CBD: 53.29 mg/ml<br><br>Oil 2 (source: product label)<br>THC: 15 mg/bottle<br>CBD: 800 mg/bottle
Skin-based applications:<br>☐ Cutaneous<br><br>☐ Transdermal	☐ Topical (lotion, cream, balm, salve)<br><br>☐ Transdermal patch				

**Note: although route of administration data not used in cannabinoid exposure calculations, it can be useful for qualitative descriptions of cannabinoid product use across a sample*

Step 3: Review Frequency and Amount of Use Data & Determine Calculation Approach

Using information provided about use, determine which approach to use for each product:

- Discrete Use Approach:
    - Use when an individual reports how frequently they use a product, and the discrete amount of product used each time
      Flower 1, Flower 2, Oil 1, Capsule

- Total Use Approach:
    - Use when an individual reports the total amount of product they use in a given time period
      Oil 2

Step 4: Convert Cannabinoid Information to Preferred Units (if necessary)

- Flower, vape cartridge OR other combustible products:
  - If necessary, convert mL vape cartridge to g (e.g., 1000mL = 1g or 500mL = 0.5g)
  - If necessary, convert % THC and %CBD to mg/g
    - Multiple percentage by 10 (e.g., 1% = 10mg/g)
  - Convert THC & THC_A to Max THC
    - *Max THC (mg) = (0.877 x THC_A mg/g) + THC mg/g*
  - Convert CBD & CBD_A to Max CBD
    - *Max CBD (mg) = (0.877 x CBD_A mg/g) + CBD mg/g*

- Oil, tincture, solutions OR topicals:
  - If necessary, convert mg/fl oz to mg/ml (*1 fl oz = 29.57ml)*

Organize Final THC and CBD Content:

Flower 1 ("Inhaled 1"):
Max THC = (.877 x 209) +0 = **183.29 mg/g THC**
Max CBD = (.877 x 10) + 10 = **18.77 mg/g CBD**

Flower 2 ("Inhaled 2") :
4% THC = 4 mg/g THC; 21.3% THC_A = 213mg/g THC_A
0.1% CBD = 1mg/g CBD; 0% CBA_A = 0mg/g CBD_A
Max THC = (.877x 213) + 4 = **190.80 mg/g THC**
Max CBD = (.877 x 0) + 1 = **1 mg/g CBD**

Capsule* ("Oral 1"):
0.3 mg/pill THC
8.1 mg/pill CBD

Oil 1* ("Mucosal 1"):
1.63 mg/ml THC
53.29 mg/ml CBD

Oil 2* ("Mucosal 2"):
15 mg THC/100ml bottle
800 mg CBD/100ml bottle

**No conversion needed*

Step 5: Discrete Use Approach: Calculate THC and CBD Exposure

Concept: Average THC or CBD Exposure = (cannabinoid content x amount used x frequency of use x duration of use)/time frame

a. Calculate THC exposure for each product:

Formula: Average THC exposure (mg/week) = $\frac{\text{mg of THC}}{\text{unit (g,mg,ml,etc.)}} \text{ x } \frac{\text{amount (g,mg,ml,etc.)}}{\text{use}} \text{ x } \frac{\text{\# of uses}}{\text{day}} \text{ x } \frac{\text{\# of days used}}{\text{wk}} \text{ x } \frac{\text{\# of weeks product was used}}{\text{total \# of weeks in interval}}$

Discrete Use THC Exposure:

$THC_{Inhaled\ 1}$ = Avg THC exposure from Flower 1 = $\frac{183.29 \text{ mg THC}}{\text{g}} \text{ x } \frac{0.25\text{g}}{\text{use}} \text{ x } \frac{1 \text{ use}}{\text{day}} \text{ x } \frac{0.75\ days^{*}}{\text{wk}} \text{ x } \frac{5.42 \text{ wks}}{12 \text{ wks}}$ = **15.52 mg THC/week**

$THC_{Inhaled\ 2}$ = Avg THC exposure from Flower 2 = $\frac{190.80 \text{ mg THC}}{\text{g}} \text{ x } \frac{0.25\text{g}}{\text{use}} \text{ x } \frac{1 \text{ use}}{\text{day}} \text{ x } \frac{0.25\ days^{*}}{\text{wk}} \text{ x } \frac{5.42 \text{ wks}}{12 \text{ wks}}$ = **5.39 mg THC/week**

$THC_{Oral\ 1}$ = Avg THC exposure from Capsule = $\frac{0.3 \text{ mg THC}}{\text{pill}} \text{ x } \frac{1 \text{ pill}}{\text{use}} \text{ x } \frac{1 \text{ use}}{\text{day}} \text{ x } \frac{7 \text{ days}}{\text{wk}} \text{ x } \frac{4.28 \text{ wks}}{12 \text{ wks}}$ = **0.75 mg THC/week**

$THC_{Mucosal\ 1}$ = Avg THC exposure from Oil 1 = $\frac{1.63 \text{ mg THC}}{\text{mL}} \text{ x } \frac{0.5 \text{ mL}}{\text{use}} \text{ x } \frac{2 \text{ uses}}{\text{day}} \text{ x } \frac{7 \text{ days}}{\text{wk}} \text{ x } \frac{5.71 \text{ wks}}{12 \text{ wks}}$ = **5.43 mg THC/week**

**Reported as 0.75 and 0.25 days/week to account for patient's use of flower 1 75% of the time and flower 2 25% of the time*

b. Calculate CBD exposure for each product:

Formula: Average CBD exposure (mg/week) = $\frac{\text{mg of THC}}{\text{unit (g,mg,ml,etc.)}} \text{ x } \frac{\text{amount (g,mg,ml,etc.)}}{\text{use}} \text{ x } \frac{\text{\# of uses}}{\text{day}} \text{ x } \frac{\text{\# of days used}}{\text{wk}} \text{ x } \frac{\text{\# of weeks product was used}}{\text{total \# of weeks in interval}}$

Discrete Use CBD Exposure:

$CBD_{Inhaled\ 1}$ = Avg CBD exposure from Flower 1 = $\frac{18.77 \text{ mg CBD}}{\text{g}} \text{ x } \frac{0.25\text{g}}{\text{use}} \text{ x } \frac{1 \text{ use}}{\text{day}} \text{ x } \frac{0.75\ days^{*}}{\text{wk}} \text{ x } \frac{5.42 \text{ wks}}{12 \text{ wks}}$ = **1.58 mg CBD/week**

$CBD_{Inhaled\ 2}$ = Avg CBD exposure from Flower 2 = $\frac{1 \text{ mg CBD}}{\text{g}} \text{ x } \frac{0.25\text{g}}{\text{use}} \text{ x } \frac{1 \text{ use}}{\text{day}} \text{ x } \frac{0.25\ days^{*}}{\text{wk}} \text{ x } \frac{5.42 \text{ wks}}{12 \text{ wks}}$ = **0.03 mg CBD/week**

$CBD_{Oral\ 1}$ = Avg CBD exposure from Capsule = $\frac{8.1 \text{ mg CBD}}{\text{pill}} \text{ x } \frac{1 \text{ pill}}{\text{use}} \text{ x } \frac{1 \text{ use}}{\text{day}} \text{ x } \frac{7 \text{ day}}{\text{wk}} \text{ x } \frac{4.28 \text{ wks}}{12 \text{ wks}}$ = **20.22 mg CBD/week**

$CBD_{Mucosal\ 1}$ = Avg CBD exposure from Oil 1 = $\frac{53.29 \text{ mg CBD}}{\text{mL}} \text{ x } \frac{0.5 \text{ mL}}{\text{use}} \text{ x } \frac{2 \text{ uses}}{\text{day}} \text{ x } \frac{7 \text{ day}}{\text{wk}} \text{ x } \frac{5.71 \text{ wks}}{12 \text{ wks}}$ = **177.50 mg CBD/week**

**Reported as 0.75 and 0.25 days/week to account for patient's use of flower 1 75% of the time and flower 2 25% of the time*

Step 6: Total Use Approach: Calculate THC and CBD Exposure

<u>Concept:</u> Average THC or CBD Exposure = cannabinoid content x (total amount used/time frame)

Note: Choose *one* formula to calculate THC and CBD exposure for each product, or adjust to desired time frame

a. Calculate THC exposure for each product:

Formula: **Average THC exposure (mg/week) =** $\frac{\text{mg of cannabinoid}}{\text{unit (g,mg,ml,etc)}} \times \frac{\text{total amount used during interval (g,mg,ml,etc.)}}{\text{\# of weeks in interval}}$

Total Use THC Exposure:

$THC_{Mucosal\ 2}$ = Avg THC exposure from Oil 2 = $\frac{\text{15 mg THC}}{\text{100 mL}} \times \frac{\text{250 mL}}{\text{12 weeks}}$ = **3.13 mg THC/week**

b. Calculate CBD exposure for each product:

Formula: **Average CBD exposure (mg/week) =** $\frac{\text{mg of cannabinoid}}{\text{unit (g,mg,ml,etc)}} \times \frac{\text{total amount used during interval (g,mg,ml,etc.)}}{\text{\# of weeks in interval}}$

Total Use CBD Exposure:

$CBD_{Mucosal\ 2}$ = Avg CBD exposure from Oil 2 = $\frac{\text{800 mg CBD}}{\text{100 mL}} \times \frac{\text{250 mL}}{\text{12 weeks}}$ = **166.67 mg CBD/week**

Step 7: Sum Exposure for Products within Each Route of Administration

Concept: Average THC or CBD Exposure= sum of the average cannabinoid exposure for each route of administration

a. Calculate THC exposure for each route of administration (inhaled, oral, mucosal, and skin-based applications)

Formulae:

THC Exposure for **Inhaled** Products: $THC_{Inhaled\ 1} + THC_{Inhaled\ 2} + THC_{Inhaled\ 3} ... + THC_{Inhaled\ n}$ = 15.52 + 5.39 = **20.91 mg THC/week**

THC Exposure for **Oral** Products: $THC_{Oral\ 1} + THC_{Oral\ 2} + THC_{Oral\ 3} ... + THC_{Oral\ n}$ =**0.75 mg THC/week**

THC Exposure for **Mucosal** Products: $THC_{Mucosal\ 1} + THC_{Mucosal\ 2} + THC_{Mucosal\ 3} ... + THC_{Mucosal\ n}$ =5.43 + 3.13 = **8.56 mg THC/week**

THC Exposure for **Skin-based** Applications: $THC_{Skin\ 1} + THC_{Skin\ 2} + THC_{Skin\ 3} ... + THC_{Skin\ n}$ = N/A

b. Calculate CBD exposure for each route of administration (inhaled, oral, mucosal, and skin-based applications)

Formulae:

CBD Exposure for **Inhaled** Products: $CBD_{Inhaled\ 1} + CBD_{Inhaled\ 2} + CBD_{Inhaled\ 3} ... + CBD_{Inhaled\ n}$ = 1.58 + .03 = **1.61 mg CBD/week**

CBD Exposure for **Oral** Products: $CBD_{Oral\ 1} + CBD_{Oral\ 2} + CBD_{Oral\ 3} ... + CBD_{Oral\ n}$ =**20.22 mg CBD/week**

CBD Exposure for **Mucosal** Products: $CBD_{Mucosal\ 1} + CBD_{Mucosal\ 2} + CBD_{Mucosal\ 3} ... + CBD_{Mucosal\ n}$ =177.50+166.67=**344.17 mg CBD/week**

CBD Exposure for **Skin-based** Applications: $CBD_{Skin\ 1} + CBD_{Skin\ 2} + CBD_{Skin\ 3} ... + CBD_{Skin\ n}$ = N/A

OPTIONAL Step 8: Sum Exposure Across Product Types

Concept: Total Average THC or CBD Exposure= sum of the average cannabinoid exposure for all products used

a. Calculate THC exposure for all products (mg/week)

Formula: Total THC Exposure = $THC_{Inhaled} + THC_{Oral} + THC_{Mucosal} + THC_{Skin}$

TOTAL THC EXPOSURE:

Total THC Exposure = 20.91 mg/week + 0.75 mg/week + 8.56mg/week = **30.22 mg THC/week**

b. Calculate CBD exposure for all products (mg/week)

Formula: Total CBD Exposure = $CBD_{Inhaled} + CBD_{Oral} + CBD_{Mucosal} + CBD_{Skin}$

TOTAL CBD EXPOSURE:

Total CBD Exposure = 1.61mg/week + 20.22 mg/week + 344.17mg/week = **366.00 mg CBD/week**

***Disclaimer**: Step 8 is included for instances where a single metric of cannabinoid exposure is needed. It is important to disclose when using this overall estimate of total exposure, particularly if multiple routes of administration are used, as differences in bioavailability are not accounted for, likely rendering this calculation less accurate than individual estimates of exposure for each route of administration (which are calculated in Step 7).*

***Note**: To calculate estimates of total cannabinoid exposure across a group of patients or participants, use Step 8 to calculate total exposure for each individual first; then calculate the average cannabinoid exposure across the group. Do NOT calculate group averages in step 7 and sum exposure for each route of administration to generate total average cannabinoid exposure.*
